# An Enhanced Ensemble Deep Neural Network Approach for Elderly Fall Detection System Based on Wearable Sensors

**DOI:** 10.3390/s23104774

**Published:** 2023-05-15

**Authors:** Zabir Mohammad, Arif Reza Anwary, Muhammad Firoz Mridha, Md Sakib Hossain Shovon, Michael Vassallo

**Affiliations:** 1 Department of Computer Science and Engineering, Bangladesh University of Business and Technology, Dhaka 1216, Bangladesh; zabir@bubt.edu.bd; 2 School of Computing, Edinburgh Napier University, Edinburgh EH10 5DT, UK; 3 Department of Computer Science, American International University—Bangladesh (AIUB), Dhaka 1229, Bangladesh; firoz.mridha@aiub.edu (M.F.M.); sakib.aiub.cs@gmail.com (M.S.H.S.); 4 Royal Bournemouth Hospital, Bournemouth BH7 7DW, UK; michael.vassallo@uhd.nhs.uk

**Keywords:** deep learning, fall detection, pre-fall detection, ensemble architecture, convolutional neural network, recurrent neural network

## Abstract

Fatal injuries and hospitalizations caused by accidental falls are significant problems among the elderly. Detecting falls in real-time is challenging, as many falls occur in a short period. Developing an automated monitoring system that can predict falls before they happen, provide safeguards during the fall, and issue remote notifications after the fall is essential to improving the level of care for the elderly. This study proposed a concept for a wearable monitoring framework that aims to anticipate falls during their beginning and descent, activating a safety mechanism to minimize fall-related injuries and issuing a remote notification after the body impacts the ground. However, the demonstration of this concept in the study involved the offline analysis of an ensemble deep neural network architecture based on a Convolutional Neural Network (CNN) and a Recurrent Neural Network (RNN) and existing data. It is important to note that this study did not involve the implementation of hardware or other elements beyond the developed algorithm. The proposed approach utilized CNN for robust feature extraction from accelerometer and gyroscope data and RNN to model the temporal dynamics of the falling process. A distinct class-based ensemble architecture was developed, where each ensemble model identified a specific class. The proposed approach was evaluated on the annotated SisFall dataset and achieved a mean accuracy of 95%, 96%, and 98% for Non-Fall, Pre-Fall, and Fall detection events, respectively, outperforming state-of-the-art fall detection methods. The overall evaluation demonstrated the effectiveness of the developed deep learning architecture. This wearable monitoring system will prevent injuries and improve the quality of life of elderly individuals.

## 1. Introduction

Falls are a major health concern for elderly individuals, with the potential to result in severe injuries, hospitalization, and even death when they cause head-related injuries. Moreover, medical studies have shown that severe injuries caused by falls are highly head related injuries. As a result, individuals must receive medical attention as soon as possible when they fall. However, detecting falls before they occur may play a critical role in preventing such incidents and improving the quality of life for seniors [1,2]. In recent years, there has been a growing interest in developing fall detection systems (FDS) that use wearable sensors and deep learning (DL) algorithms to detect and alert caregivers or medical professionals in the case of a fall.

There is a rapid growth in the use of wearable sensors for fall detection systems, using inertial sensors such as accelerometers, gyroscopes, magnetometers, and inertial measurement units (IMU), as well as pressure sensors [3,4]. Several studies have demonstrated the effectiveness of using accelerometers on the waist and wireless accelerometers in identifying fall events with high accuracy rates ranging from 86% to 99% [5,6]. Considerable efforts have been devoted to creating wearable devices that can detect falls after they occur to ensure prompt medical assistance. Since it only provides assistance after the impact injuries have already occurred, it is considered reactive. Therefore, researchers have now redirected their focus toward fall prevention systems (FPS), which are performed through fall risk assessment and intervention. This approach can filter out older individuals with high fall risks early and apply appropriate strategies to prevent future falls [7,8]. A Pre-Fall prediction is an emerging approach that can overcome the limitations of fall detection systems. The approach can also help prevent fall-related injuries by predicting falls before they occur and by activating on-demand fall safety systems, such as wearable airbags, in real-time. However, accurately predicting falls before the ground impact is challenging due to the short duration of falls (around 0.8 s) and extensive annotation of the falling process. Very little research has been conducted under such circumstances. Musci et al. [9] developed an RNN-based model with LSTM blocks on the SisFall dataset, achieving sensitivity scores of 0.93, 0.84, and 0.93 for Non-Fall, Pre-Fall, and Fall events, respectively, as well as specificity scores of 0.95, 0.95, and 0.98, and accuracies of 0.94, 0.89, and 0.95. However, the sensitivity and accuracy scores needed improvement in their approach. Torti et al. [10] also used the SisFall dataset and developed an embedding RNN method, obtaining sensitivity scores of 0.88, 0.91, and 0.97 for Non-Fall, Pre-Fall, and Fall events, specificity scores of 0.97, 0.90, and 0.97, and accuracies of 0.92, 0.90, and 0.97. Unfortunately, this model also struggled to achieve good sensitivity and specificity scores for Non-Fall events. In contrast, Xiaoqun et al. [11] proposed a better model, ConvLSTM, based on convolution and the LSTM network. This model achieved a comparatively better performance, with sensitivity scores of 0.93, 0.93, and 0.96, specificity scores of 0.96, 0.94, and 0.98, and accuracies of 0.94, 0.93, and 0.97 for Non-Fall, Pre-Fall, and Fall events, respectively. While existing approaches have shown promising results, there are still some limitations that need to be addressed. More research is required to improve them, as there has been adequate study of this three-class problem.

For gait analysis, Anwary et al. [12] investigated the optimal location for wearable sensors, Ref. [13] automated the extraction of gait parameters, and [14] evaluated gait abnormalities. In this paper, we proposed a class-based ensemble architecture approach that encourages learning each ensemble for one specific event to improve the accuracy of elderly fall detection. Our approach utilizes wearable sensors placed on the waist to capture multiple sources of information and increase the robustness of the system. We evaluated our proposed method on a real-world dataset and compared its performance with existing state-of-the-art approaches, demonstrating its potential for real-world deployment in elderly care facilities and home settings.

The following are the contributions of the proposed work:

- The proposed work introduces a wearable fall detection framework that can accurately detect Non-Fall, Pre-Fall, and Fall events. In the case of a Pre-Fall event, the framework will activate safety measures to prevent severe head injuries before the individual hits the ground. Additionally, the framework will issue remote notifications in the event of a fall to ensure timely medical assistance.
- A novel class-based ensemble architecture was developed for receiving sensory data through a head model, resulting in improved accuracy due to every ensemble memorizing a single class.
- We conducted experiments on different configurations of the proposed architecture’s CNN and RNN components. By combining the use of CNN and RNN, our model can capture both short-term and long-term dependencies in human motion. The CNN model focuses on short-term dependencies, while the ensemble RNN model captures long-term dependencies by analyzing feature maps over a sequence of data. Our result analysis confirms that this architecture provides higher accuracy than other models.

## 2. Materials and Methods

### 2.1. Dataset and Material

Many human activity datasets with fall events have been proposed in the literature [15]. However, most of them have been simulated by young volunteers, which is challenging for real-life scenarios. We chose six datasets from among them for consideration: [16,17,18,19,20,21]. Following a comparative analysis shown in Table 1, we decided that SisFall [21] was the most suitable dataset for this work. We selected SisFall for developing and evaluating deep learning algorithms for two major reasons. First, compared to other publicly available datasets such as MobiFall [18] and UMAFall [19], it has the largest amount of data in terms of the number of subjects and the number of activities. Second, there are 15 older subjects out of a total of 38 subjects in the SisFall dataset with same number of male and female participants, and the protocol is validated by medical staff. As a result, the data pattern in the SisFall dataset should be similar to that of the real-life activities of daily living (ADLs) and fall scenarios of older people. ADLs are routine activities that individuals perform every day to take care of themselves, such as bathing, dressing, grooming, toileting, eating, and mobility.

**Figure 1:**
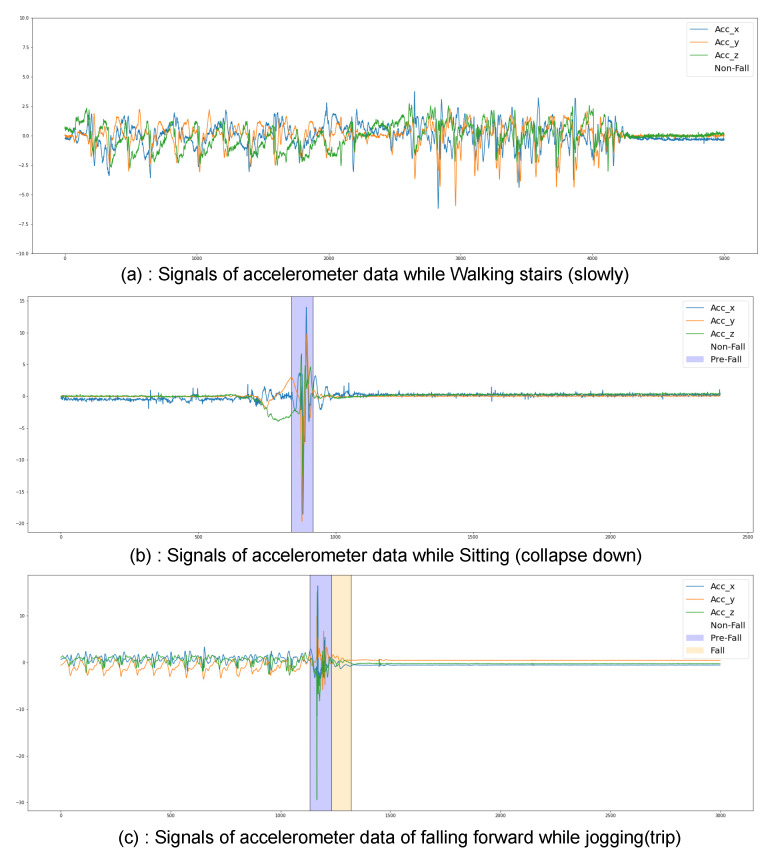
The figure illustrates the annotation of the SisFall dataset’s accelerometer sensor data [21] of samples. (**a**) consists of a Non-Fall state (no fall has occurred). (**b**) consist of Non-Fall and Pre-Fall states (fall is initiated but did not impact the ground). (**c**) consist of Non-Fall, Pre-Fall, and Fall states (fall is initiated and impacts the ground).

The SisFall dataset contains recordings from 38 volunteers: 23 young subjects and 15 elderly subjects, who each performed 34 different activities in a controlled scenario (19 ADLs and 15 falls) with multiple trials for a total of 4510 complete sequences. The SisFall dataset was collected using a custom board with two tri-axial accelerometers and a tri-axial gyroscope operating at 200 Hz. The recording device was placed on the volunteers’ waists with a fixed orientation to the body. Annotations in the SisFall dataset classify each entire activity as a fall or an ADL. However, there is no specific indication of when a fall occurs in the sequence of readings or when a particular ADL occurs, which is insufficient to support the training of an RNN method aiming for the real-time detection of falls. Therefore, the annotations must have been presented in event-specific time intervals to accommodate the training process. A labeling proposal was introduced to this dataset in [9]. Each temporary sample was categorized as either belonging to a fall event, an alert (fall hazard), or a daily life activity. To the best of our knowledge, this is the only publicly available fall dataset that takes into account fall hazard events, such as the moments just before a fall or a dangerous situation when a user was able to avoid falling. Figure 1 illustrates a reflective diagram of annotations, which shows the three-axis acceleration data of different samples.

In [9]’s annotation process of SisFall, each raw SisFall sequence was first passed through a low-pass Butterworth filter to remove high-frequency noise. Then, using standard Z-Score normalization, all readings were converted into the [−1, +1] range. As a recurrent neural network requires a fixed length of sequences, this length is known as width. The dimension of each value in the sequence is fixed. The values in this issue are a tuple with three elements that correspond to the three axes of the accelerometer or gyroscope. From now on, we will refer to each tuple with the term sample throughout the manuscript. Similarly, each sequence of samples with a fixed width will be referred to as a block. The dataset establishes a block width of 256 timesteps and approximately 1.28 s of sensor data. A 50 percent overlapping was used to extract the block from the whole sequence of raw sensor data. Each block must have an associated label corresponding to the event class to train an RNN mode. Therefore, we applied the proposal from [9], in which each block is categorized based on the frequency of appearance of the most relevant class. The labeling data are associated with three types of events:

- Non-fall: the time interval during which the person performs ADLs.
- Pre-fall: the time interval during which a person transitions from a controlled to a dangerous state, which may result in a fall.
- Fall: the time interval during which the person is in a state transition that leads to a fall.

### 2.2. Proposed FDS Framwork

**Figure 2:**
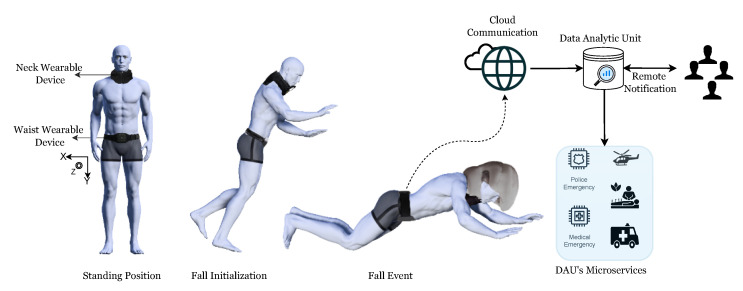
The figure illustrates the proposed fall detection framework. Two interconnected wearable devices have been attached to the subject: one placed on the waist is an embedded device for data acquisition from sensors and fall detection, and the other is an airbag helmet designed to inflate to prevent head-related injuries when falls are detected. Additionally, it issues a remote notification to provide immediate medical assistance.

The proposed fall detection framework, as depicted in Figure 2, aims to detect falls and prevent fall-related injuries in elderly individuals by utilizing wearable devices. The framework consists of two interconnected devices that can communicate with each other through wired or Bluetooth connections. The first device is an embedded wearable sensor placed on the waist, which contains an accelerometer and a gyroscope for data acquisition and fall detection. The second device is an airbag helmet worn around the neck to prevent head injuries resulting from a fall.

The initial phase of the proposed framework involved the acquisition of sensor data through the embedded wearable device in real-time. To prepare the data for processing, normalization was applied to normalize the sensor data within the range of [−1, +1]. The normalized data were then passed to a deep learning model, as discussed in Section 2.4, to perform a three-class classification.

In the event that the deep learning model predicts a Pre-Fall event, the embedded device sends a signal to the airbag helmet to inflate, thereby preventing head injuries. Similarly, if a Fall event is predicted, the model sends an alert to the cloud via Cloud Communication, which exploits backhaul networks to transmit data to the cloud. The Data Analytics Unit (DAU) in the cloud provides various microservices, such as sending remote notifications to relatives and arranging emergency medical services for initial medical aid.

It should be noted that while, the proposed fall detection framework has been designed and its functionality has been described, the wearable devices and their hardware implementation have not yet been developed as part of this study. The proposed framework represents a concept that will require further development and implementation to realize its full potential.

### 2.3. Architectural Motivation

Ensemble approaches in neural network architecture have been found to enhance model performance by combining several sub-models to improve accuracy [22]. Compared to simple models, ensemble architectures are less susceptible to overfitting and produce more accurate results [23]. Additionally, ensembles are capable of dealing with complex data patterns that a single model may struggle to recognize [24]. Ensemble methods have been implemented in various domains such as geospatial land classification, face recognition, image segmentation, and more. In literature [25], the authors addressed three key reasons why ensemble architectures may outperform traditional models. Firstly, the training phase may not contain sufficient data to produce the best classifier. Secondly, a single algorithm may fail to converge to the global optimum, but an ensemble of algorithms starting from different points could lead to a better approximation of the global optimum. Finally, the space being searched may not contain any optimum position, but an ensemble may lead this space to a better optimum position. Ensembles are a powerful tool in machine learning, and researchers continue to explore new ways to leverage their potential for improved model performance.

In the current context of deep learning, there exist three widely used types of ensemble classifier architecture: (1) stacked ensembles, (2) weight average ensembles, and (3) class-based ensembles. The stacked ensemble architecture involves input data passing through multiple sub-models, and the data stream is then directed to a final learning model which generates the output of the classifier. This process can be represented mathematically as

(1) $$E_{\phi }\left(x\right)=f\left(\varepsilon_{1}\left(x\right),\varepsilon_{2}\left(x\right),\cdot \cdot \cdot \cdot \cdot \cdot ,\varepsilon_{n}\left(x\right)\right)$$

where *x* is input data, $E_{\phi }\left(\right)$ is a stacked ensemble model, ε() is the ensemble sub-models, f() is the final learning model, and *n* is the number of sub models.

The weight average ensemble architecture calculates the predictions of multiple models separately and then combines them through weight multiplication calculations to generate the final prediction [26]. This can be expressed mathematically as

(2) $$E_{\phi }\left(x\right)=argmax\left(\omega_{1}\cdot \varepsilon_{1}\left(x\right),\omega_{2}\cdot \varepsilon_{2}\left(x\right),\cdot \cdot \cdot \cdot \cdot \cdot ,\omega_{n}\cdot \varepsilon_{n}\left(x\right)\right),$$

where *x* is input data, $E_{\phi }\left(\right)$ is a weighted average ensemble model, ε() is the ensemble sub-models, ω is weights for each ensemble model and *n* is the number of sub models.

The class-based ensemble architecture is characterized by having the same number of ensemble models as the number of classes to be identified. Each ensemble model is trained to recognize a specific category, and together they form a complete system that can accurately classify input data [27]. This architecture can be mathematically derived as follows:

(3) $$E_{\phi }\left(x\right)=argmax\left(\varepsilon_{1}\left(x\right),\varepsilon_{2}\left(x\right),\cdot \cdot \cdot \cdot \cdot \cdot ,\varepsilon_{n}\left(x\right)\right),$$

where *x* is input data, $E_{\phi }\left(\right)$ is a class-based ensemble model, ε() is an ensemble sub-model for the i-th class, and *n* is the number of output classes.

Inspired by the enhancements achieved through ensemble architectures, we have created an ensemble architecture that delivers comparable results to the class-based ensemble architecture. However, our proposed architecture has some slight variations from the class-based ensemble architecture. Mathematically, the proposed architecture can be formulated as follows:

(4) $$E_{\phi }\left(x\right)=argmax\left(\varepsilon_{1}\left(f(x)\right),\varepsilon_{2}\left(f(x)\right),\cdot \cdot \cdot \cdot \cdot \cdot ,\varepsilon_{n}\left(f(x)\right)\right),$$

where *x* is input data, $E_{\phi }\left(\right)$ is the proposed ensemble model, ε() is the ensemble sub-model for the i-th class, f() is the head model, and *n* is the number of output classes.

**Figure 3:**
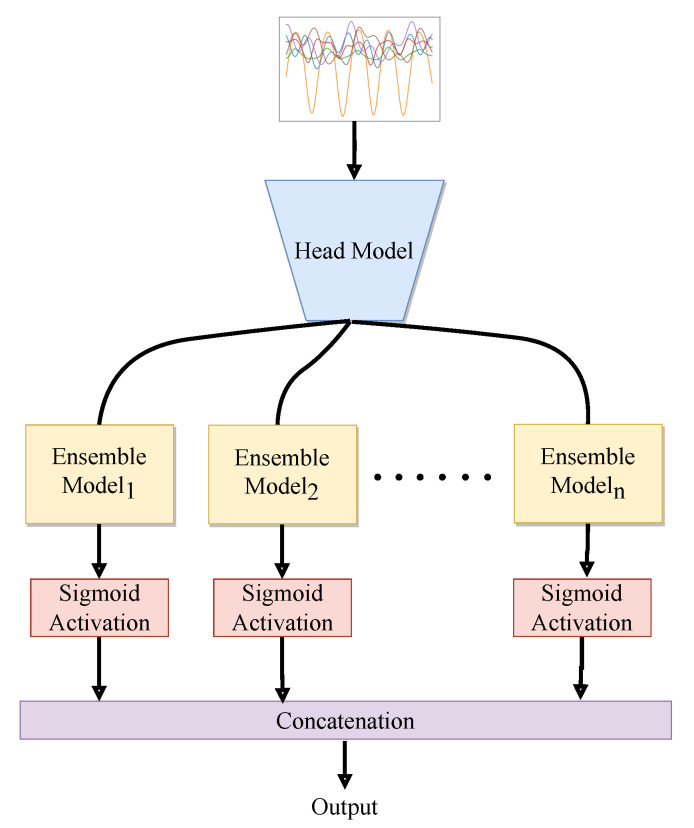
The figure depicts the architectural strategy of the class-based ensemble architecture. Inputs flow through the head model, which is further passed through the class-specific ensemble submodels.

Our proposed ensemble architecture was inspired by the success of class-based ensembles but has some unique features [28]. Instead of passing inputs directly to the ensemble models, our architecture uses a head model as an auxiliary feature extractor. The head model selects relevant feature embeddings and reduces the dependence on the ensemble models. We kept the number of ensembles equal to the number of target classes, and each ensemble model learns explicitly to identify a particular class. Figure 3 illustrates the proposed ensemble approach. This approach allows individual ensemble models to focus on recognizing a specific class, which may improve the accuracy of the overall proposed architecture.

### 2.4. Architecture

The proposed architecture comprises two main components: the feature extractor model (head model) and the ensemble model. The feature extractor model receives input sensory data and generates embeddings, which are then fed to the ensemble models. Each ensemble model is responsible for recognizing a specific target class, so the number of ensemble models must match the number of possible categories. Using the head model offers several advantages over the typical class-based model. Higher parameters in an architecture increase the risk of overfitting, which is especially problematic for wearable devices that require less parameterized models. Passing the input through a head model reduces the number of irrelevant features and the number of trainable parameters, leading to a significant reduction in computation and overfitting [29]. Overall, our proposed ensemble architecture offers the following benefits: each ensemble extracts only the necessary information to recognize a specific class, resulting in an approximate optimal position for each class and superior accuracy; and the use of the head model significantly reduces the number of parameters required for each ensemble model.

**Figure 4:**
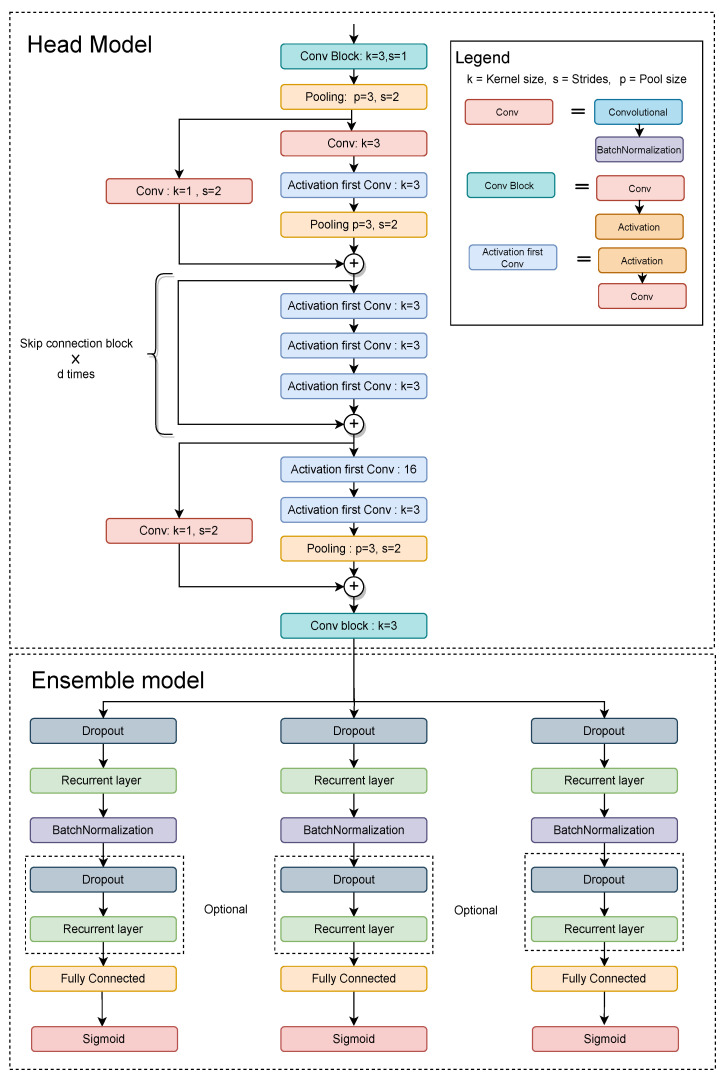
The figure illustrates the head and ensemble model of the architecture. The head network receives input sensor data, and the processed data. It further forwards the processed data to the multiple ensemble models. The optional block provides flexibility as it allows the user to choose how many times to use it, or to not use it at all.

Figure 4 illustrates the architectural specifications of the proposed class-based ensemble architecture. The architecture is split into two parts: the head model and the ensemble model. The head model consists of CNN and the ensemble model consists of RNN. The use of combined CNN and RNN was inspired by [11] work. CNN acts as a feature extractor that provides an abstract representation of the input sensor data in a feature map. The CNN captures the short-time dependency of the sensor data. Further, the feature maps are passed through an ensemble model. The recurrent layers of the ensemble models deal with long-term temporal dynamics of the activation of the feature maps and recognize features over time in sequential data. CNN layers capture features from raw data and pass them to RNN layers to discover temporal correlations, potentially saving computing time.

### 2.5. Data Augmentation

Data augmentation has been proven to enhance the generalization of machine learning models, and is especially effective when training deep neural networks. It can be defined as a technique to enhance the diversity of the data by minor transformations of already existing data. In addition, data augmentation helps to prevent overfitting and improve the generalization ability of a DL model. In image recognition, data augmentation is a well-known process for training DL models. However, augmenting wearable sensor data is challenging due to maintaining label-preserving augmentation. Label-preserving augmentation refers to a technique used in machine learning to augment the training data by applying transformations to the input images while preserving the labels. For example, scaling the acceleration data may change their labels because the motion intensity differentiates some labels. However, small changes in magnitude may preserve the labels. Nevertheless, in this work, we used arbitrary rotation, scaling, and jitter transformation processes defined in [30] to augment the data. Furthermore, the transformation process was labeled invariant, which does not change the sensor data characteristics. Thus, we maintained the label of the augmented data based on their original label. The transformations are follows:

- Rotation: Rotation of sensor data refers to the transformation of the data by rotating it around a specified axis or point in three-dimensional space. The rotation can be represented by three angles, known as Euler angles, which specify the amount of rotation around each axis. In this experiment, we uses a rotation angle in the range (−π,π) radian.
- Scaling: Scaling sensor data refers to rescaling the magnitude of the sensor data in a window by multiplying it by a random scalar, where the random scalar is sampled from a normal distribution with a mean of 1 and a standard deviation of 0.1. The choice of a standard deviation of 0.1 for the scaling factor helps to ensure that the augmented data are not too different from the original data, while still introducing some variability
- Jitter: Jittering is a data augmentation technique used to simulate sensor noise. It adds random noise to the sensor data to make it more robust against both additive and multiplicative noise. By adding noise to the data, the model can learn to be more resilient to unexpected variations in the sensor readings. In this case, the standard deviation of the noise added was set to 0.01, which determines the amount of random noise that is added to the sensor data during the jittering process.

Most use data augmentation processes to train neural networks to increase the data samples. However, our study used a non-generative augmentation (online augmentation) process to train the network. The process can be seen as randomly taking training data and applying augmentation techniques before fitting the model. This technique does not save any disk data and preprocesses data in real-time while making the training batch data.

Algorithm 1 outlines the process for generating each batch of data during training of the neural network model. The algorithm starts by initializing transformation probabilities for rotation, scaling, and jitter. For each batch, the algorithm selects half of the batch’s data and randomly chooses a transformation for each data point based on the assigned probabilities, and applies the chosen transformation to the selected data within the batch. This process was repeated for each batch during the training phase, with the objective of enhancing the model’s resilience to noise and augmenting its overall performance.   
**Algorithm 1:** Online data augmentation algorithm.
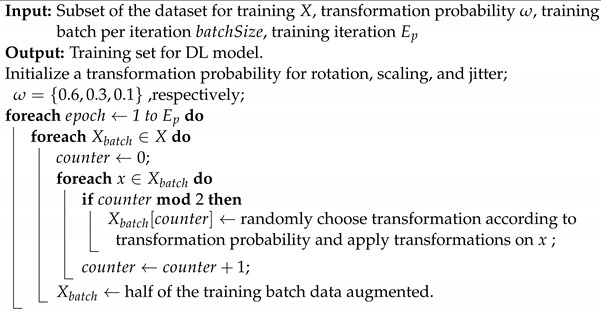


## 3. Results

### 3.1. Evaluation Metrics

Due to the highly imbalanced data of each class, traditional classification accuracy is not suitable for analyzing the system’s effectiveness. To measure the effectiveness of the proposed architecture, we calculated the metrics individually for each event, where Events∈{Non−Fall,Pre−Fall,Fall}. That allowed us to compute accuracy using an unbalanced number of Non-Fall, Pre-Fall, and Fall events in a single test. Three evaluation metrics were used in the method, which is presented as follows:

- Accuracy: Accuracy is one of the most fundamental evaluation metrics. It can be formally defined as the ratio of accurateness over all experiments. It can be defined as:
  (5) $$Accuracy_{c\in \mathrm{Events}}=\frac{TP_{c}+TN_{c}}{TP_{c}+TN_{c}+FP_{c}+FN_{c}}.$$
- Sensitivity: Sensitivity is also known as recall score or true positive rate. It refers to the correctness of the true positive events of each available class. It can be mathematically defined as
  (6) $$Sensitivity_{c\in \mathrm{Events}}=\frac{TP_{c}}{TP_{c}+FN_{c}}.$$
- Specificity: Specificity is also known as the true negative rate. It refers to the percentage of all negative samples that the model correctly predicts as negative. It can be represented as
  (7) $$Specificity_{c\in \mathrm{Events}}=\frac{TN_{c}}{TN_{c}+FP_{c}},$$

where c∈Events and Events→{Non−Fall,Pre−Fall,Fall}. For a specific event, *c*, $TP_{c}$ is the number of events correctly classified as the event; $FN_{c}$ is the number of events incorrectly classified as the event; $FP_{c}$ is the number of events misclassified as the event; and $TN_{c}$ is the number of events correctly classified as not the event.

### 3.2. Model’s Training

The overall architecture was implemented using the Tensorflow, Keras, Scikit-learn, and NumPy libraries. The input data for training have six dimensions, including a three-axis accelerometer and a three-axis gyroscope. The architecture was trained using a batch size of 128 with a maximum epoch of 200. With an initial learning rate of 0.0005, the Adam [31] optimizer was used to train the architecture and the loss function used was weighted binary cross-entropy loss.

A particular train test set was selected to access the proposed model’s generalization and evaluate the architectures with the state-of-the-art. There were 23 young and 15 elderly volunteers who contributed to the dataset. To mitigate any bias in the architecture and ensure accurate measurement of each architecture’s accuracy, we defined the test set containing four young and two elderly people’s activities as in [9]. The remaining person’s activities were split into the training and validation sets with an 80–20% ratio. This train–test set separation prevented us from having the same subject appear in both the training and test sets. Thus, we could measure the architecture’s robustness in a real-life scenario. All the experiments were conducted on this specific dataset. Furthermore, the final displayed result represents three training runs’ mean and standard deviation.

### 3.3. Result Analysis

In the result comparison, we split the section into three subsections. First, we analyzed the proposed architecture with different hyperparameters, such as the number of skipped convolution blocks, the number of recurrent layers, and the width of the network, in terms of the sensitivity of the architecture. Secondly, we analyzed the baseline architecture from hyperparameter tuning with different convolution layers, such as convolutions and separable convolutions [32], pooling layers, such as max-pooling and average pooling, activation layers, such as Relu and Swish [33], and recurrent layers, such as LSTM, GRU [34], and bidirectional [35]. Finally, we compared our proposed architectural results with state-of-the-art real-time FDS detection works.

Table 2 summarizes the result of the hyperparameter tuning experiment on testing data. The proposed architecture receives the sensory data through the head model. The head model is used as a feature extractor of the input data to generate sequential embedding. Furthermore, the embeddings are passed to the ensemble model for sequential processing to classify events. As the experiments show, increasing the number of skipped convolution blocks 3 to 4 in the head model decreases the architecture’s performance. The ensemble model consists of recurrent layers for processing sequential data. Increasing the number of recurrent layers from 1 to 2 in the ensemble model increases the architecture’s performance by 2%. Increasing the number of recurrent layers from 2 to 3 has a performance gain of 0.05%. However, increasing the recurrent layers results in a considerable parameter expansion, and sequential processing requires time. As our process is in real-time, the increasing processing time is not considered for the work. Our experiments found that three skipped convolution blocks for the head model and two recurrent layers for the ensemble model are suitable for the architecture.

Traditional deep learning architecture increases the width of the network for each block. However, our experiment shows that the same architectural width produces better results than increasing the width of the architecture. Furthermore, the optimal width of the network is set at 16. Table 2 shows that expanding the network’s width decreases the architecture’s performance because, in the ensemble model, the width is greater than the data’s sequentiality. However, the low-level width of the network has one other advantage; the model is less parameterized and requires fewer computational costs.

We further investigated our proposed baseline architecture in the table with different layer configurations. Table 3 shows the performance analysis with varying compositions of layers. First, the head model was analyzed using two different convolutions, pooling, and activation functions: the general convolution and the separable convolution with the ReLU and the Swish activation functions, along with max pooling and average pooling. Max pooling retains the most prominent features of the feature map and average pooling retains the average values of features of the feature map. Due to the high peaks of sensor data during the fall, the max pool works better than the average pool because it helps the architecture preserve the sensor data’s peak values. From Table 3, we saw that the swish activation function gives a superior performance boost of 3% to the ReLU activation function in all different configurations. Swish is a smooth continuous function that allows a small number of negative weights to be propagated through the network while ReLu thresholds all negative weights to zero. Additionally, the trainable parameters allow better tuning of the function to maximize information propagation and push for a smoother gradient. It helps to optimize the network to be more accessible, thus generalizing better and faster.

On the other hand, compared to general convolution with swish activation, separable convolution with swish activation gives a more acceptable result, with a performance gain of 1.5%. The reason lies in the configuration of separable convolution. While standard convolution computes channel-wise and spatially in a single phase, separable convolution divides the calculation into two steps: depthwise convolution uses a single convolutional filter for each channel. Moreover, pointwise convolution generates a linear combination of the depthwise convolution output. Therefore, separable convolutions work on multiple filters simultaneously and tend to understand the information characteristics better. Additionally, separable convolution provides better nonlinearity than traditional convolutions, with excellent convergence speed, minor accuracy gain, and fewer trainable parameters than standard convolution.

Secondly, the ensemble model was analyzed using three types of recurrent layers: LSTM, GRU, and bidirectional. From Table 3, we chose LSTM as the baseline recurrent layer in our proposed architecture. Although bidirectional LSTM achieved a performance gain of 0.5% over LSTM, bidirectional recurrent layers increase the network’s computational complexity and memory occupancy, making such performance improvements not worthwhile for real-time. We also considered GRU in our experiment. However, due to the absence of the output gate, GRU required slightly fewer computations and parameters than LSTM but entailed a reduction in sensitivity of over 4%. From the experiment, we found that a dropout rate of 0.5 was suitable for our architecture.

Table 4 presents a comparison of our proposed architecture with the state-of-the-art systems. The results demonstrate that our proposed architecture outperforms the state-of-the-art in all aspects. Two literature reviews [10,11] represent the current state-of-the-art, respectively. Ref. [10] achieves mean accuracies of 0.93, 0.90, and 0.97 for Non-Fall, Pre-Fall, and Fall events, respectively. Ref. [11] outperformed this result using a convolutional recurrent architecture with mean accuracies of 0.94, 0.93, and 0.97. However, our proposed ensemble-based convolutional recurrent architecture achieves an even higher accuracy, with mean accuracies of 0.95, 0.97, and 0.99 for all classes.

**Figure 5:**
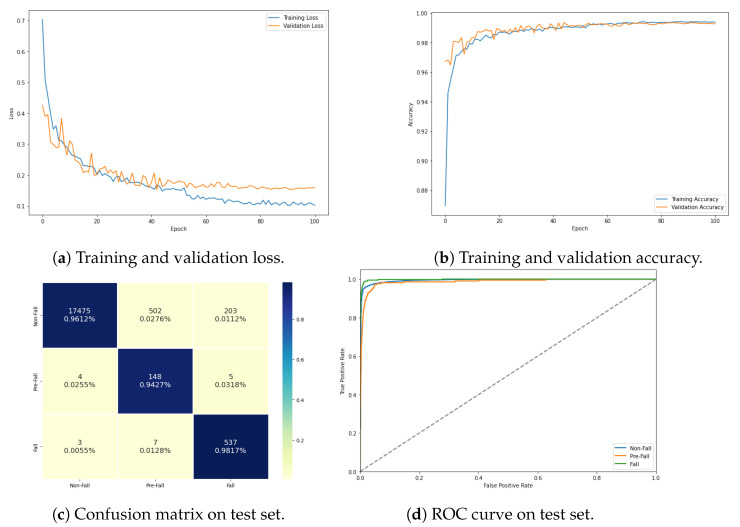
The figure represents the (**a**) training and validation loss, (**b**) training and validation accuracy, (**c**) confusion matrix, and (**d**) ROC curve of the resultant architecture.

In terms of specificity, our proposed architecture achieves a mean specificity of 0.98, 0.97, and 0.99 for Non-Fall, Pre-Fall, and Fall events, respectively, which is higher than that of the state-of-the-art architectures. With respect to sensitivity, our proposed ensemble model achieves a mean sensitivity of 0.96, 0.94, and 0.98, which is higher than that of the state-of-the-art systems’ sensitivity; Ref. [10]’s 0.88, 0.91, and 0.97 and [11]’s 0.93, 0.93, and 0.96 for Non-Fall, Pre-Fall, and Fall events, respectively. Figure 5 illustrates the losses, accuracy, confusion matrix, and ROC curve of the resultant architecture, respectively. Figure 5a and b indicate that the model did not overfit during training, as the training and validation loss decreased consistently and the training and validation accuracy increased consistently without diverging from each other. However, it should be noted that these graphs are based on the performance of the deep learning model on simulated data, and the actual hardware implementation of the model has not been developed in the current scope of the study. Therefore, the results of the model’s performance on real-world data may differ from the simulated data presented in the figure.

## 4. Discussion

In this study, we proposed a class-based enhanced ensemble deep-learning architecture that utilizes accelerometer and gyroscope sensor data to classify Pre-Fall and Fall events in elderly individuals. The results indicate that our ensemble architecture outperforms the state-of-the-art models in terms of sensitivity, specificity, and overall accuracy to identify Pre-Fall, Fall, and Non-Fall events. The SisFall dataset and real-world scenarios predominantly contain Non-Falls, making the detection of Fall instances challenging. However, our proposed architecture achieves a higher classification sensitivity in the Non-Fall class, resulting in a lower false alarm rate and a 3% improvement. Even though a 3% improvement in accuracy may seem small, in the context of an automated fall monitoring system for the elderly, it can have significant practical benefits. It can lead to more reliable and timely alerts, minimize false positives and unnecessary anxiety, and provide an added layer of safety through Pre-Fall detection and the activation of safety mechanisms to prevent or minimize fall-related injuries. Our proposed ensemble architecture comprises individual models that focus on recognizing specific events, enabling them to achieve their ideal state of identifying a particular event while disregarding others. This unique feature has the potential to improve the accuracy of the architecture. Additionally, the combination of convolutional and recurrent layers enhances the architecture’s performance by capturing short-term dependencies of human motion through CNN and long-term dependencies of feature maps over the time domain in the sequential data through RNN. We also used online data augmentation to enhance the network’s performance during training. This method involves augmenting half of the existing data during each training iteration, increasing the data diversity, and improving the architecture’s accuracy. Furthermore, we employed separable convolution to enhance the performance of the architecture. Unlike standard convolution, separable convolution divides the calculation into depth-wise and pointwise convolution steps, providing better nonlinearity and improving convergence speed. Swish activation is another technique that we used, allowing negative weights to propagate through the network and improving performance. Finally, we utilized LSTM with memory cells to ease the learning of long-term time dependence on motion data by learning to store and output information based on training. Our proposed hybrid deep learning ensemble approach using wearable sensors is a promising technique for enhancing elderly fall detection. It outperforms the state-of-the-art models, achieves high accuracy, and has the potential to improve the lives of elderly individuals by providing timely fall detection and intervention.

## 5. Conclusions

In this study, we proposed a deep learning ensemble algorithm to enhance fall detection for elderly people using wearable sensors. The proposed method uses a class-based ensemble architecture that combines convolutional and recurrent neural networks. Our approach achieved a mean accuracy of 95%, 0.96%, and 0.98% for Non-Fall, Pre-Fall, and Fall events, respectively. Moreover, the proposed method outperformed existing fall detection methods in terms of sensitivity and specificity. The results demonstrate that the proposed algorithm is highly effective in detecting Pre-Fall and Fall events using wearable sensors. Therefore, our approach can be implanted into wearable inertial sensor-based systems to predict Fall events in real-time, allowing protective devices to be triggered in time to prevent fall-related injuries in the case of Pre-Fall events and issue a remote notification for timely medical assistance in the case of Fall events.

The findings of this research have significant implications for improving the safety and well-being of elderly people. Falls are a significant public health problem that can lead to serious injuries and even death in the elderly population. The proposed method offers a potential solution to address this issue by providing accurate and timely fall detection using wearable sensors. The proposed algorithm’s high performance in detecting Pre-Fall and Fall events can help prevent fall-related injuries and improve the quality of life for elderly individuals. In conclusion, our study provides a novel approach to fall detection and offers a promising solution for improving the safety and well-being of elderly individuals.

## Figures and Tables

**Table 1:** Comparative analysis of existing fall detection datasets.

Dataset	Type of Sensors	Sensor Position	Number of Subject (Male/Female)	Age Range	Trials	Number of Activity Types (ADL/Fall)	Number of Samples (ADL/Fall)
Vilarinho et al. [16]	Accelerometer, Gyroscope and Megnetometer.	Wrist, thigh pocket	3	22–32	-	22 (7/15)	117 (45/72)
T-fall [17]	Accelerometer	Thigh pocket	10 (7/3)	20–42	3	-	10,909 (9883/1026)
Mobi-fall [18]	Accelerometer, Gyroscope	Thigh pocket	24 (17/7)	22–47	1/3/6	13 (9/4)	630 (342/288)
Uma-fall [19]	Accelerometer, Gyroscope and Magnetometer	Waist, wrist, ankle and chest.	17 (11/6)	18–55	3	11 (8/3)	561 (332/209)
Umi-shar [20]	Accelerometer	Thigh pocket	30 (6/24)	18–60	-	17 (9/8)	11,771 (7579/4192)
Sis-fall [21]	Accelerometer and Gyroscope	waist	38: 15 elderly, 23 young adults	18–75	1/5	34 (19/15)	4505 (2207/1798)

**Table 2:** The table illustrates the hyperparameter tuning experiment, such as the number of skipped convolution blocks, the width of the head model, the number of recurrent layers, and the width of the ensemble network, in terms of the sensitivity of the architecture. Table is based on all possible outcomes of the best results obtained from hyperparameter tuning. We performed extensive experimentation with various hyperparameter configurations to find the optimal settings for our ensemble deep neural network architecture. As mentioned in the paper, our proposed model is an ensemble of convolutional neural network (CNN) and recurrent neural network (RNN), which is a nonlinear model.

Head Model		Ensemble Model		Sensitivity		
No. of skipped Convolutional Blocks	Width	No. of Recurrent layers	Width	Non-Fall	Pre-Fall	Fall
2	(16,16,16)	1	(16)	0.89	0.87	0.94
2	(16,16,32)	1	(32)	0.88	0.85	0.91
2	(16,16,16)	2	(16,16)	0.90	0.88	0.94
2	(16,16,32)	2	(32,64)	0.88	0.87	0.91
2	(16,16,16)	3	(32,32,64)	0.88	0.88	0.90
2	(16,32,64)	3	(64,64,128)	0.87	0.86	0.89
2	(16,16.16)	4	(16,16,32,32)	0.89	0.88	0.90
2	(16,16,32)	4	(32,32,64,64)	0.89	0.82	0.88
3	(16,16,16)	2	(16,16)	0.91	0.89	0.96
3	(16,32,32)	2	(32,32)	0.90	0.87	0.94
3	(16,32,64)	3	(64,64,128)	0.90	0.88	0.94
3	(16,16,16)	3	(16,16,16)	0.91	0.89	0.97
3	(16,16,32)	4	(32,32,64,64)	0.91	0.87	0.94
3	(16,32,64)	4	(64,64,128,128)	0.90	0.88	0.93
4	(16,16,16)	2	(16,16)	0.92	0.90	0.94
4	(16,16,32)	2	(32,64)	0.90	0.89	0.92
4	(16,32,64)	3	(64,64,128)	0.89	0.87	0.89
4	(16,16,32)	3	(32,32,64)	0.90	0.88	0.90
4	(16,16,16)	4	(16,16,16,16)	0.92	0.90	0.95
4	(16,32,64)	4	(64,64,128,128)	0.89	0.88	0.91

**Table 3:** The table illustrates the performance comparison with varying compositions of layers.

Head Model			Ensemble Model		Sensitivity		
Convolutions	Pooling	Activations	Recurrent layers	Dropout Rate	Non-Fall	Pre-Fall	Fall
Conv	Max	ReLU	LSTM	0.5	0.91	0.89	0.97
Conv	Max	ReLU	GRU	0.25	0.90	0.89	0.93
Conv	Max	ReLU	Bi-directional	0.8	0.91	0.89	0.94
Conv	Max	Swish	LSTM	0.8	0.92	0.87	0.96
Conv	Max	Swish	GRU	0.5	0.90	0.88	0.94
Conv	Max	Swish	Bi-directional	0.25	0.93	0.89	0.97
Conv	Average	ReLU	LSTM	0.5	0.89	0.87	0.93
Conv	Average	ReLU	GRU	0.25	0.88	0.84	0.89
Conv	Average	ReLU	Bi-directional	0.8	0.90	0.83	0.85
Conv	Average	Swish	LSTM	0.25	0.90	0.90	0.90
Conv	Average	Swish	GRU	0.5	0.88	0.86	0.90
Conv	Average	Swish	Bi-directional	0.8	0.90	0.89	0.91
SeparableConv	Max	ReLU	LSTM	0.8	0.91	0.89	0.96
SeparableConv	Max	ReLU	GRU	0.25	0.90	0.88	0.95
SeparableConv	Max	ReLU	Bi-directional	0.5	0.92	0.90	0.96
SeparableConv	Max	Swish	LSTM	0.5	0.96	0.94	0.98
SeparableConv	Max	Swish	GRU	0.25	0.92	0.88	0.95
SeparableConv	Max	Swish	Bi-directional	0.8	0.95	0.91	0.98
SeparableConv	Average	ReLU	LSTM	0.5	0.89	0.85	0.91
SeparableConv	Average	ReLU	GRU	0.25	0.87	0.80	0.90
SeparableConv	Average	ReLU	Bi-directional	0.5	0.89	0.85	0.91
SeparableConv	Average	Swish	LSTM	0.8	0.88	0.86	0.92
SeparableConv	Average	Swish	GRU	0.25	0.87	0.83	0.87
SeparableConv	Average	Swish	Bi-directional	0.5	0.89	0.86	0.90

**Table 4:** The table illustrates the comparison of proposed framework with the state-of-the-art.

Metrics	Events	[9]	[10]	[11]	Proposed
Sensitivity	Non-Fall	0.93	0.88	0.93	0.96
	Pre-Fall	0.84	0.91	0.93	0.94
	Fall	0.93	0.97	0.96	0.98
Specificity	Non-Fall	0.95	0.97	0.96	0.98
	Pre-Fall	0.95	0.90	0.94	0.97
	Fall	0.98	0.97	0.98	0.99
Accuracy	Non-Fall	0.94	0.92	0.94	0.95
	Pre-Fall	0.89	0.90	0.93	0.97
	Fall	0.95	0.97	0.97	0.99

## Data Availability

Not applicable.

## Abbreviations

The following abbreviations are used in this manuscript:

FDS	Fall Detection Systems
DL	Deep Learning
IMU	Inertial Measurement Units
FPS	fall prevention systems
RNN	Recurrent Nueral Network
LSTM	Long-Short Term Memory
CNN	convolutional Neural Network
ADL	Activity of Daily Livings
GRU	Gated Recurrent Unit
DAU	Data Analytics Unit
